# NPS-1034 Exerts Therapeutic Efficacy in Renal Cell Carcinoma Through Multiple Targets of MET, AXL, and TNFRSF1A Signaling in a Metastatic Model

**DOI:** 10.3390/cells13201713

**Published:** 2024-10-17

**Authors:** Ya-Chuan Chang, Chien-Te Liu, Chia-Ying Yu, Wen-Wei Sung

**Affiliations:** 1 School of Medicine, Chung Shan Medical University, Taichung 40201, Taiwan; raptor7037@gmail.com (Y.-C.C.); s1001020@gm.csmu.edu.tw (C.-T.L.); cyyu2015@gmail.com (C.-Y.Y.); 2 Department of Urology, Chung Shan Medical University Hospital, Taichung 40201, Taiwan; 3 Institute of Medicine, Chung Shan Medical University, Taichung 40201, Taiwan

**Keywords:** kidney cancer, lung metastasis, tumor necrosis factor receptor 1, tyrosine kinase inhibitor, systemic treatment

## Abstract

Renal cell carcinoma (RCC) has diverse pathological subtypes, most of which have a poor prognosis. Patients with advanced RCC require systemic therapies for disease control. Although targeted therapies and immune checkpoint inhibitors have shown therapeutic efficacy, patients eventually succumb to disease progression. Therefore, additional therapies targeting different pathways are needed to provide more therapeutic options for sequential treatment. Our study explored the biological mechanisms and therapeutic outcomes for NPS-1034, a dual MET/AXL inhibitor, in RCC, both in vivo and in vitro. Our results showed that NPS-1034 can significantly inhibit tumor proliferation and induce cancer cell apoptosis. Besides MET and AXL, known targets of NPS-1034, we identified TNFRSF1A as another target gene inhibited by NPS-1034 via antibody arrays. This was further supported by next-generation sequencing, showing that the TNF signaling pathway is one of the most significant NPS-1034-regulated pathways. Furthermore, one of the identified target genes, GADD45A, responsible for NPS-1034 anticancer properties, was significantly associated with patient survival in RCC. GADD45A expression was significantly upregulated via NPS-1034 and downregulated via TNFRSF1A overexpression. Finally, its therapeutic efficacy was demonstrated in vivo, showing that NPS-1034 significantly alleviated the tumor burden and inhibited cell proliferation in a lung metastatic animal model. In conclusion, we explored the therapeutic mechanism of NPS-1034 and found that it targets not only MET and AXL but also TNFRSF1A. In a lung metastatic animal model, we confirmed that NPS-1034 is a potential candidate for systemic therapy in RCC.

## 1. Introduction

Renal cell carcinoma (RCC) is one of the most lethal common urologic cancers. It accounts for 2.2% of all adult malignancies in global cancer statistics, with diagnoses made primarily between 55 and 75 years of age and a male-to-female incidence of 6.1 to 3.2, respectively. It is now among the top ten most frequently diagnosed malignancies [1,2,3,4,5,6]. Clear cell RCC (ccRCC) represents the most common histopathological diagnosis among RCC cancer-related deaths. ccRCC originates primarily from proximal convoluted tubules and is one of the most vascular malignancies. ccRCC is more aggressive than other common subtypes of RCC, but with doublet or triplet immunotherapy combined with/without targeted molecular therapies, patients can achieve an improved prognosis [7,8,9,10,11,12,13,14]. However, patients eventually succumb to disease progression owing to inevitable drug resistance, and more therapies targeting different pathways are needed to provide improved therapeutic options for sequential treatment.

Given the characteristic hematogenous route of metastatic spread of RCC, it has been postulated that the primary angiogenesis inducer in ccRCC is vascular endothelial growth factor (VEGF). Von Hippel–Lindau (VHL) could regulate the expression of VEGF, binding to at least one related VEGF receptor (VEGFR). VEGFRs have become therapeutic targets against RCC, including using VEGF inhibitors, mTOR inhibitors, and other tyrosine kinase inhibitors (TKIs) [15,16]. Multiple signaling pathways are activated via VEGFR phosphorylation, including the Raf-MEK-Erk and PI3K/AKT/mTOR pathways, which are induced by MET and AXL. The VEGF inhibitors MET and AXL have demonstrated significant activity as first- and second-line treatments for metastatic RCC. The VEGF-targeting monoclonal antibody bevacizumab has been studied both as a single agent and in combination with chemotherapeutic agents (e.g., pazopanib, sunitinib, and cabozantinib), with modest results [17,18,19,20]. Notably, 10–20% of patients fail to respond to antiangiogenic therapy, and the remaining patients show tumor growth after a median of 10–12 months of treatment. Discontinuing treatment with TKI leads to accelerated endothelial cell proliferation and the rapid recurrence of angiogenesis. Few patients achieve complete remission, and the median death from metastatic ccRCC occurs 2 years after diagnosis. Owing to the dependence of RCC on antiangiogenic therapy, drugs targeting MET and AXL might represent a novel therapeutic approach [15,21,22].

NPS-1034 inhibits multiple receptor tyrosine kinases (RTKs) implicated in tumor growth, metastasis, and angiogenesis, with targets including MET and AXL [23]. Both MET and AXL RTKs are located upstream of multiple biological reaction processes. AXL regulates cell responses, including cell survival, proliferation, migration, and phagocytosis, which may affect the downstream PI3K/Akt and Janus kinase–STAT signaling pathways. Regulating AXL is crucial for tumor development and neovascularization and is an important therapeutic target for metastatic cancer cells [24,25,26,27]. Moreover, abnormal MET activation is found in many cancers through gene mutation, amplification, and protein overexpression. Therefore, MET imbalance is closely related to tumor growth, invasion, and metastasis [27,28,29,30]. Given the potential for a dual MET and AXL inhibitor to minimize the resistance seen to date with antiangiogenic agents, we aimed to investigate the anticancer efficacy and underlying mechanisms of NPS-1034, both in vitro and in vivo, to explore the potential value of alternative adjuvant therapy in RCC.

## 2. Methods

### 2.1. Cell Culture

In the present study, we used three RCC cell lines, including A498 (JCRB 1388, Japan), 786-0 (JCRB 1397, Osaka, Japan), and Caki-1 (ATCC^®^ HTB-46™, Virginia, VA, USA), to perform our experiments. According to the provider’s protocol, the three cell lines were cultured in standard medium (Eagle’s minimum essential medium, #41500-034, Gibco, Grand Island, NY, USA or RPMI 1640 medium, #31800-022, Gibco, Waltham, MA, USA) supplemented with 10% fetal bovine serum (#A52567-01, Gibco, Waltham, MA, USA) and maintained at 37 °C in an incubator containing 5% CO_2_. Both standard media were supplemented with 100 μg/mL streptomycin (#15140-122, Gibco, Waltham, MA, USA), 100 U/mL penicillin (#15140-122, Gibco, Waltham, MA, USA), 0.1 mM NEAA (#11140-050, Gibco, Waltham, MA, USA), 1 mM sodium pyruvate (#11360-070, Gibco, Waltham, MA, USA), and NaHCO_3_ (#S5761, Sigma-Aldrich, Burlington, MA, USA) as described [31,32,33].

### 2.2. MTT Assay

We performed an MTT assay in triplicate to assess the cytotoxicity and drug effect of NPS-1034 (CAS: 1221713-92-3, purity: ≥98.0%, MedChemExpress, Monmouth Junction, NJ, USA) on the RCC cell lines. First, we seeded 1 × 10^4^ cells/well of RCC cells into a 96-well plate and incubated it overnight for cell attachment. After treatment with NPS-1034 (0, 10, 20, 40, 80, and 160 μM) for 24 h, we removed the drug-containing medium and added 100 μL of 0.5 mg/mL MTT solution (#M5655, Sigma-Aldrich, Burlington, MA, USA) for 3 h of incubation. Then, we replaced the medium with 100 μL of DMSO to solubilize the formazan crystals, and the optical density was subsequently read using an ELISA reader (#Multiskan FC, Thermo Scientific, Waltham, MA, USA) at a wavelength of 570 nm [32,33].

### 2.3. Colony Formation Assay

The proliferation abilities of the RCC cell lines were assessed in triplicate with a colony formation assay. RCC cells including A498 (1000 cells/well), 786-0 (500 cells/well), and Caki-1 (500 cells/well) were seeded in 6-well plates and incubated overnight for cell attachment. After being treated with NPS-1034 (0, 20, and 80 μM) for 24 h, the cells were incubated in drug-free medium for 9 days. After 9 days of cultivation, the cells were carefully rinsed with phosphate-buffered saline (PBS) twice. The colonies were then fixed with 95% ice-cold ethanol for 20 min, followed by staining with 0.5% crystal violet (#C3886, Sigma-Aldrich, Burlington, MA, USA) for 15 min. The colonies were photographed, and the number of colonies was counted [32,33].

### 2.4. Flow Cytometry

To determine the effect of NPS-1034 on the distribution of RCC cells in their cell cycle phases, a flow cytometry analysis was performed using a flow cytometer (FACSCantoTMII Cell Analyzer, BD Biosciences, Vienna, Austria). In brief, RCC cells were treated with NPS-1034 for 48 h and fixed in 70% ice-cold ethanol followed by staining with 4 μg/mL propidium iodide (PI) (#P4170, Sigma-Aldrich, Burlington, MA, USA) and 0.5 mg/mL of DNase-free RNase A (#11119915001, Sigma-Aldrich, Burlington, MA, USA) in PBS. Afterward, the cell cycle phase distribution was measured with a flow cytometer, and charts were depicted with FlowJo software (version 10.10, BD Biosciences, Vienna, Austria). For cell apoptosis analysis, we utilized the Annexin V-FITC/PI double-staining kit (Strong Biotech Corporation, Taipei, Taiwan) to stain the apoptotic cells; then, we calculated the cell apoptotic rate of each group using a flow cytometer. According to the manufacturer’s protocol, the RCC cells were suspended in 100 μL of binding buffer and 2 μL of Annexin V-FITC/PI followed by incubation in the dark for 15 min. Subsequently, the double-stained cells were assessed and analyzed via flow cytometry. All experiments were performed in triplicate [32,33].

### 2.5. Hoechst 33342 Staining

Since chromatin condensation and the appearance of apoptotic bodies are special features of apoptosis, we used the Hoechst 33342 nuclear-staining reagent (#H3570, Invitrogen, Waltham, MA, USA) to identify the percentage of apoptosis in each group of RCC cells under the NPS-1034 treatment. The morphological changes in the nuclei at different concentrations of NPS-1034 were assessed using fluorescence microscopy (ImageXpress PICO, San Jose, CA, USA). In brief, RCC cells were seeded in a 6-well plate with a density of 1.5~3.5 × 10^5^ cells/well and exposed to NPS-1034 (0, 20, and 80 μM) for 24 h, followed by drug-free incubation for another 24 h. Afterward, the cells were stained with 10 μg/mL of Hoechest-33342 for 20 min in an incubator at 37 °C. Then, we carefully rinsed the cells with PBS and photographed them via fluorescence microscopy (excitation wavelength, 350–390 nm; emission wavelength, 420–480 nm). The apoptotic rate and condensed nuclei were calculated from 5 random visual fields selected in each group [32,33].

### 2.6. Human Apoptosis Array for Proteome Profiling

Using a human Proteome Profiler™ Antibody Array (#ARY009, R&D Systems, McKinley Place NE, Minneapolis, MN, USA), the relative expression levels of 35 apoptosis-linked proteins were detected in duplicate based on the antibodies embedded on the array’s nitrocellulose membrane. For sample preparation, A498, 786-0, and Caki-1 cells were treated with NPS-1034 (0 and 80 μM) for 48 h and harvested for cell lysate extraction based on the manufacturer’s protocol. In total, 400 μg of protein was immersed in each array membrane. Then, the membranes were washed and incubated with horseradish peroxidase-conjugated secondary antibodies, followed by a chemireagent. The staining intensity was quantified using an ImageQuant LAS4000 instrument (GE Healthcare, Chicago, IL, USA), and the integrated density was analyzed using ImageJ (version 1.54j, National Institutes of Health, Bethesda, MD, USA) [32,33].

### 2.7. RNA Sequencing

For sample preparation, RCC cell lines were treated with NPS-1034 (0 and 80 μM) for 48 h, and the total RNA was extracted using the TRIzol^®^ Reagent (#15596026, Invitrogen, Waltham, MA, USA) according to the instruction manual. Library preparation, sequencing, alignment, and differential expression analyses were performed by Genomics (Taipei, Taiwan) according to their official protocol. In summary, genes with a *p*-value ≤ 0.05 and ≥ 2-fold changes were considered significantly differentially expressed. Overrepresented Gene Ontology (GO) and Kyoto Encyclopedia of Genes and Genomes (KEGG) pathway enrichment analysis compared cluster functions in the R package cluster profiler. The top 10 most enriched GO terms and KEGG pathways were visualized with a *p*-value <  0.05 cut-off criterion. Finally, the most enriched pathway-related genes were identified for subsequent analysis.

### 2.8. TNFRSF1A Overexpression Transfection

Based on the most enriched pathway-related genes identified from the RNA sequencing results, we selected several possible target genes and used a lentiviral transfection vector to change the expression of a specific gene in the cell to reverse verify the gene’s function. TNFRSF1A overexpression can be performed using TNFRSF1A (NM_001065) human-tagged ORF clone lentiviral particles (OriGene, Rockville, MD, USA). Briefly, 10^5^ cells were seeded in a 12-well plate and cultured overnight; the original medium was replaced with 1 mL of fresh medium containing 5 μg/mL polybrene the next day, and an appropriate amount of virus suspension was added. After culturing at 37 °C for 15 h, the medium was replaced with fresh medium, and culturing continued for another 24 to 48 h. We then screened the successfully transfected cells using a medium containing 2 μg/mL of puromycin. Overall, overexpression transfection revalidated the underlying pathways altered by NPS-1034, and its downstream protein expression was monitored to confirm whether it was reversed by the overexpression transfection.

### 2.9. Western Blotting

For sample preparation, the RCC cell lines were treated with NPS-1034 (0, 20, and 80 μM) for 48 h and then harvested for Western blotting. Proteins from RCC cells were extracted with RIPA lysis buffer (#89901, Thermo Scientific, Waltham, MA, USA) in the presence of 100X Protease and Phosphatase Inhibitor Single-Use Cocktail (#78442, Thermo Scientific, Waltham, MA, USA). After sonicating on ice, the lysed cells were collected via centrifugation (10,400 rpm for 20 min at 4 °C), followed by saving the supernatant for further procedures. Aliquots of lysate containing equal amounts of 30 μg proteins were loaded onto 8–15% sodium dodecyl sulfate–polyacrylamide gel electrophoresis (SDS–PAGE) and subsequently transferred to Immobilon^®^-E Transfer Membranes (#IEVH85R, Millipore, Burlington, MA, USA). The membranes were blocked at room temperature with 5% non-fat milk for 2 h and incubated (diluted 1:1000) at 4 °C overnight with primary antibodies against the following proteins: MET (A17366, ABclonal, Woburn, MA, USA), p-MET (AP0077, ABclonal, Woburn, MA, USA), AXL (A17874, ABclonal, Woburn, MA, USA), p-AXL (AF2228, Bio-Techne, Minneapolis, MN, USA), PI3K (ab191606, Abcam, Waltham, MA, USA), p-PI3K (AP0854, ABclonal, Woburn, MA, USA), AKT(ab179463, Abcam, Waltham, MA, USA), p-AKT (AP0637, ABclonal, Woburn, MA, USA), GADD45A (13747-1-AP, Proteintech, San Diego, CA, USA), TNFR1 (MAB6251, Bio-Techne, Minneapolis, MN, USA), IκBα (A11397, ABclonal, Woburn, MA, USA), p-IκBα (AP0999, ABclonal, Woburn, MA, USA), NFκB1 (A6667, ABclonal, Woburn, MA, USA), p-NFκB1 (AP0125, ABclonal, Woburn, MA, USA), p65 (A19653, ABclonal, Woburn, MA, USA), p-p65 (AP0215. ABclonal, Woburn, MA, USA), Survivin (A1551, ABclonal, Woburn, MA, USA), Claspin (A17202, ABclonal, Woburn, MA, USA), Caspase-3 (19677-1-AP, Proteintech, San Diego, CA, USA), c-Caspase-3 (#9661, Cell Signaling Technology, Danvers, MA, USA), Caspase-7 (#12827, Cell Signaling Technology, Danvers, MA, USA), c-Caspase-7 (#9491, Cell Signaling Technology, Danvers, MA, USA), PARP, and p-PARP (13371-1-AP, Proteintech, San Diego, CA, USA). The membranes were then washed with TBST buffer and incubated with the Goat Anti-Mouse (C04001, CROYEZ, Taipei, Taiwan) and Goat Anti-Rabbit (C04003, CROYEZ, Taipei, Taiwan) secondary antibodies for 1 h at room temperature. Proteins were visualized using the Immobilon^TM^-Western Chemiluminescent HRP Substrate (Millipore, Burlington, MA, USA) on the ImageQuant™ LAS4000 (GE Healthcare, Chicago, IL, USA) [32,33].

### 2.10. Animal Experiments

Specific pathogen-free 8-week-old male BALB/c mice were provided by the National Laboratory Animal Center (Taipei, Taiwan). The animal use protocol was approved by Chung Shan Medical University Experimental Animal Center (No. 2775, 01/01/23–12/31/25). All mice were maintained under the required conditions and had free access to food and water throughout the experiment. After 1 week of acclimatization (9 weeks old), the mice were randomly assigned to groups. For the control group, mouse lung metastatic renal carcinoma cells (Renca-luc) were collected and resuspended in 100 μL of saline (containing 3 × 10^5^ cells) and injected intravenously for tumor formation. The treatment group also received Renca-luc cells intravenously, and on day 21, the treatment group received low or high doses of NPS-1034 (10 or 30 mg/kg) orally and daily for 5 weeks according to previous studies [23,30]. The mice were humanely euthanized on day 56, and the tumors were excised, weighed, and stored appropriately for further analysis.

### 2.11. Histopathological Study

After animal euthanization, lungs with tumor particle tissues were dissected and embedded into paraffin blocks and then sectioned into 3 μm thick sections as blank slides. Following deparaffinization and rehydration, the tissue sections were stained with hematoxylin and eosin (Sigma-Aldrich, Burlington, MA, USA) and Ki67 (#ab15580, Abcam, Cambridge, UK) according to the manufacturer’s protocol. The stained specimens were visualized and histopathological analysis was performed using TissueFAX Plus (version 2.0, TissueGnostics, Vienna, Austria) [32,33].

### 2.12. Statistical Analysis

Statistical analysis was performed using IBM SPSS (version 20.0, Chicago, IL, USA). Data are presented as the mean ± SD. Student’s *t*-test was used for continuous or discrete data analysis to calculate the significance between different groups. All statistical tests were two-sided, and values of *p* < 0.05 were considered statistically significant (* *p* < 0.05; ** *p* < 0.01; *** *p* < 0.001) [32,33].

## 3. Results

### 3.1. NPS-1034 Blocks Proliferation of RCC Cells In Vitro

**Figure 1:**
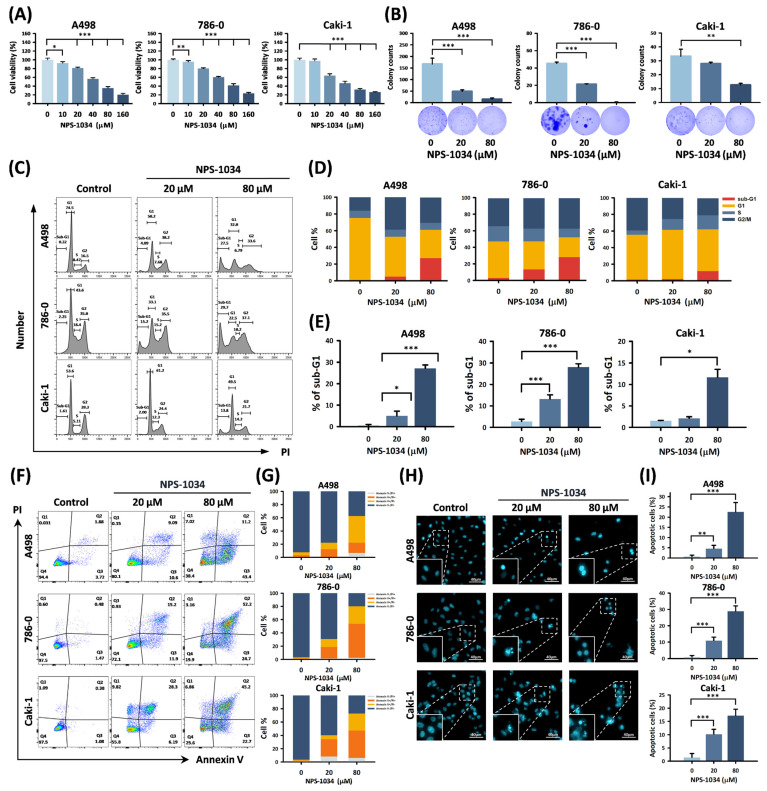
NPS-1034 suppresses cell proliferation and induces apoptosis with the accumulation of a sub-G1-phase population of RCC cell lines. (**A**) An MTT assay was performed to analyze the cell viability of RCC cell lines A498, 786-0, and Caki-1 after NPS-1034 exposure for 24 h. (**B**) A colony formation assay was performed to analyze the RCC cell lines’ clonogenic ability after NPS-1034 exposure for 24 h. (**C**) A cell cycle analysis was performed after 48 h of treatment with NPS-1034 using a flow cytometer. The proportion of the cell cycle (**D**) and the cell percentage of the sub-G1 phase (**E**) are shown. (**F**) Flow cytometry results with Annexin V-FITC/PI staining. RCC cell lines were treated with NPS-1034 for 48 h, and (**G**) the proportions of Annexin V+/−/PI+/− cells are shown. (**H**) Fluorescence images were taken after Hoechst 33342 staining. Apoptosis-associated morphological changes are emphasized by white squares, and the (**I**) bar chart shows the percentage of apoptotic cells. Data are shown as mean ± S.D. (* *p* < 0.05; ** *p* < 0.01; *** *p* < 0.001).

We aimed to evaluate the antitumor effects and clarify the potential downstream mechanisms of NPS-1034 in RCC cells with the A498, 786-0, and Caki-1 cell lines. Cell viability significantly decreased with dose-dependency after 24 h of NPS-1034 treatment, as shown in Figure 1A (all *p* < 0.001). Furthermore, the colony counts significantly decreased with NPS-1034 80 μM, as shown in Figure 1B (A498, 786-0, and Caki-1: 15.7 ± 5.0, 0.0 ± 0.0, and 12.7 ± 1.2 colony counts compared with the control group: 167.3 ± 25.5, 40.33 ± 4.51, and 33.3 ± 5.0; *p* < 0.001, *p* < 0.001, and *p* < 0.01, respectively).

### 3.2. NPS-1034 Induces Apoptosis in RCC Cells

The effects of NPS-1034 on cell cycle distribution and cell death were evaluated via flow cytometry. In the NPS-1034 treatment, the sub-G1 phase increased significantly in all three cell lines, as shown in Figure 1C–E (A498, 786-0, and Caki-1: 27.1%, 28.1%, and 11.7%, respectively, compared with the control group: 0.6%, 2.8%, and 1.6%; *p* < 0.001, *p* < 0.001, and *p* < 0.05, respectively).

Annexin V-FITC/PI double staining estimated apoptosis. From 20 and 80 μM of NPS-1034, the percentage of Annexin V+/PI− (early apoptosis) cells and Annexin V+/PI+ (late apoptosis) cells significantly increased in the A498, 786-0, and Caki-1 cells, as shown in Figure 1F,G (20 μM: 21.5%, 29.0%, and 31.9%, *p* < 0.01, *p* < 0.001, and *p* < 0.01, respectively; 80 μM: 56.0%, 77.7%, and 66.5%, *p* < 0.01, *p* < 0.001, and *p* < 0.001, respectively) compared with the control group (7.4%, 2.6%, and 1.9%, respectively).

In the NPS-1034 fluorescence microscopy Hoechst 33342 staining treatment, chromatin condensation and nuclear volume reduction increased in a high concentration range. These morphological changes were observed in nuclei in RCC cells, confirming that NPS-1034 induced cell death by promoting cell apoptosis in A498, 786-0, and Caki-1 cells (20 μM: 4.6%, 11.0%, and 10.3%, *p* < 0.01, *p* < 0.001, and *p* < 0.001, respectively; 80 μM: 22.6%, 28.9%, and 17.2%, *p* < 0.001, *p* < 0.001, and *p* < 0.001, respectively; Figure 1H,I) compared with the control group (0.7%, 0.9%, and 1.4%, respectively).

### 3.3. Identification of Apoptosis-Related Proteins Altered by NPS-1034 Treatment

To further assess the underlying molecular mechanisms of the anticancer effect of the NPS-1034 treatment on RCC cells, the human Proteome Profiler™ Antibody Array separately detected differences in 35 apoptosis-related protein expression levels in duplicate between parental cells and 48 h 80 μm NPS-1034-treated A498, 786-0, and Caki-1 cells. Comparisons of the control and quantified results are shown in Supplementary Figure S1A. As shown in Supplementary Figure S1B, the quantified results reveal that the downregulation of the upstream death receptor TNFR1 significantly decreased in RCC cells compared with the control group (43.85%, 17.64%, and 63.63%, respectively).

### 3.4. GO and KEGG Enrichment Analysis

**Figure 2:**
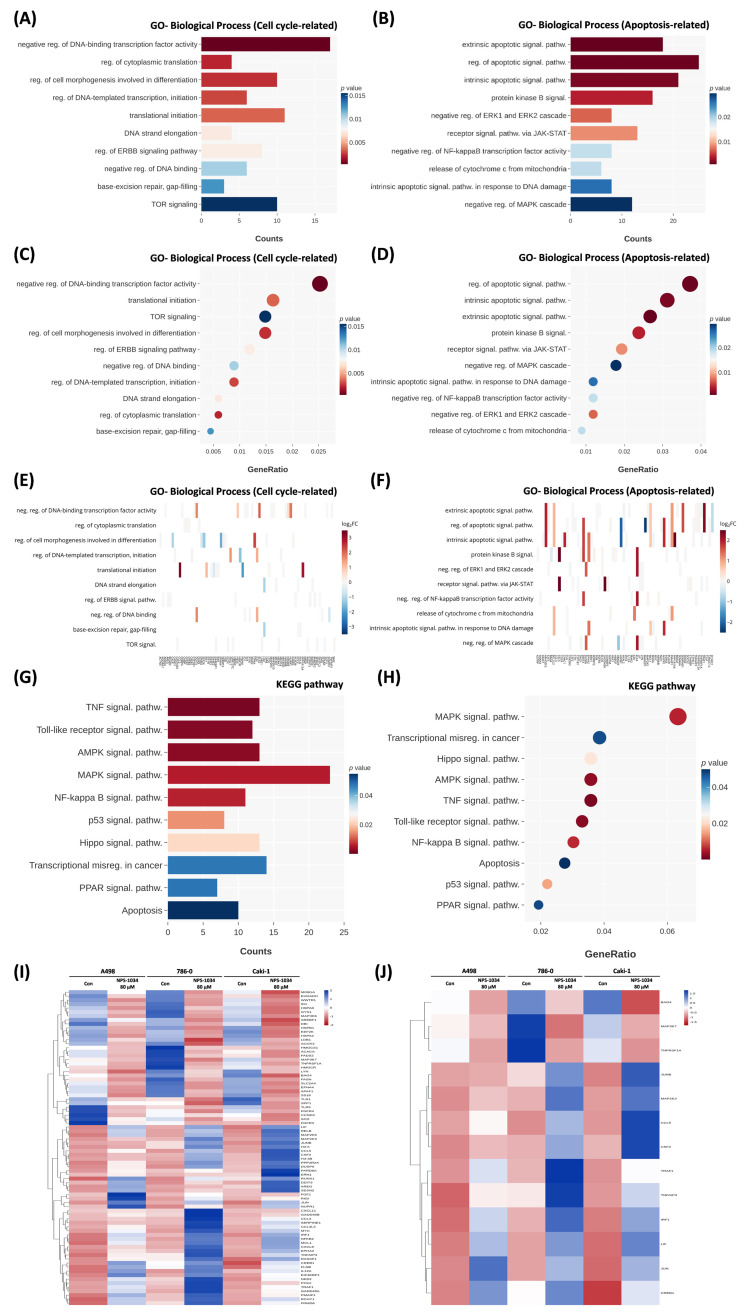
Gene ontology and KEGG enrichment analysis of NPS-1034-treated RCC cells. Biological processes enriched in relation to the (**A**,**C**,**E**) cell cycle or (**B**,**D**,**F**) apoptosis in RCC cells treated with 80 μM of NPS-1034 for 48 h. Terms in (**A**,**B**) the bar plot and (**E**,**F**) heatmap were ordered by *p*-value, while terms in the (**C**,**D**) dot plot were ordered by count number. (**G**) Dot plot and (**H**) bar plot of KEGG pathway enrichment analyses showing the identified statistically significantly enriched pathways under 80 μM of NPS-1034 for 48 h. (**I**) Heatmap analysis of the top 10 enriched pathways. Blue: upregulation; red: downregulation. (**J**) Heatmap analysis of the TNF signaling pathway revealing the clearest difference.

To elucidate the potential biological function of the 48 h 80 μM NPS-1034 treatment on RCC cells, we conducted GO and KEGG pathway enrichment analyses of RNA sequences. The GO distribution is presented in Figure 2. In terms of biological process ontology, the cell cycle and the apoptosis-related signaling pathway were significantly enriched. The most significantly enriched GO term of the cell cycle was related to the initiation of cell differentiation, transcription, and translation (negative regulation of binding transcription factor, GO: 0043433, *p* = 3.81 × 10^−4^). In contrast, the most significantly enriched GO term of the apoptotic signaling pathway was related to the initiation of apoptosis and several well-known signaling pathways, leading to apoptosis (extrinsic or intrinsic apoptotic signaling pathway, GO: 0097191, *p* = 9.81 × 10^−4^, GO: 0097193, *p* = 1.78 × 10^−3^). A heat plot shows the degree of the rise and fall of genes under the pathways related to the cell cycle or apoptosis, of which the extrinsic and intrinsic apoptotic signaling pathways changed significantly.

Additionally, the KEGG pathway analysis identified 309 signaling pathways. The top 10 most enriched pathways were visualized, as shown in Figure 2G,H. KEGG pathways were significantly enriched by the gene ratio and counts (*p*-value < 0.05), with the most significantly enriched including the TNF signaling pathway, the AMPK signaling pathway, the NF-κB signaling pathway, and the apoptosis pathways (*p* < 0.01, *p* < 0.01, *p* < 0.01, and *p* < 0.08, respectively). Mass spectrometry and heatmap analysis showed the expression profiles of the top 10 enriched pathways, as shown in Figure 2I, and effectively distinguished between the control and the 80 μM NPS-1034 treatment for RCC cells. The heatmap of the TNF signaling pathway revealed the clearest difference, as shown in Figure 2J.

### 3.5. NPS-1034 Exerts Anticancer Effects Through MET, AXL, and TNFRSF1A Signaling

**Figure 3:**
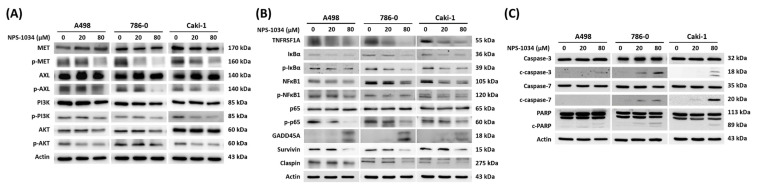
NPS-1034 activates GADD45A, inhibits the MET and TNFRSF1A downstream signaling pathway proteins’ expression level, and upregulates the pro-apoptosis protein expression level in RCC cells. (**A**) Validation of NPS-1034, an MET/AXL inhibitor, on the MET downstream pathway protein expression level in RCC cells. (**B**) Assessment of the regulatory role of GADD45A and TNFR1-related pathways, such as the IκBα/NFκB signaling pathway. (**C**) The expression levels of several pro-apoptosis proteins were detected in RCC cells treated with NPS-1034 (0, 20, and 80 μM) for 48 h via Western blotting.

To examine the underlying mechanisms, we first verified the MET, AXL, and downstream pathways and the PI3K/AKT signaling pathways. As shown in Figure 3A, the NPS-1034 treatment inhibited p-MET and p-AXL instead of total MET and AXL. Additionally, decreased p-PI3K and p-AKT were observed. Based on the array and the GO and KEGG enrichment analyses, we hypothesized that GADD45A might be responsible for the anticancer role of NPS-1034 under TNFRSF1A/IκBα/NFκB signaling. Figure 3B shows that NPS-1034 suppressed TNFRSF1A, p-IκBα, p-NFκB, and p-p65. The GADD45A, survivin, and claspin downstream target genes were also suppressed. Furthermore, we confirmed that apoptotic signaling induced via NPS-1034 increased elevated cleaved caspase7, cleaved caspase3, and cleaved PARP expression (Figure 3C).

### 3.6. TNFRSF1A Overexpression Reversed Downstream Signaling and Anticancer Effects by Inhibiting GADD45A

**Figure 4:**
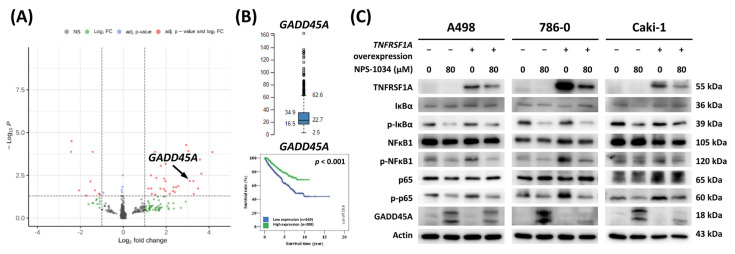
The high expression of GADD45A correlates with a favorable prognosis and effect on the survival rate of patients with RCC. TNFRSF1A overexpression reversed the anticancer effects of NPS-1034 in RCC cells. (**A**) A volcano plot showing the differential expression of genes altered by NPS-1034 treatment in RCC cells. (**B**) Relative GADD45A mRNA expression in 877 patients. Kaplan–Meier plots showing the survival probabilities of RCC patients stratified by GADD45A expression. Patient survival details were derived from TCGA. (**C**) The increases in GADD45A-related pathway expression and the decreases in IκBα/NFκB-related pathway expression are all significantly reversed via TNFRSF1A overexpression.

The differential expression genes altered by NPS-1034 are displayed in a volcano plot (Figure 4A). Among the results, the mRNA expression of growth arrest and DNA damage-inducible protein α (GADD45A) increased significantly. The mRNA expression and survival months for GADD45A in tumor tissues were obtained from 877 RCC patients in the TCGA database, as shown in Figure 4B. As shown in Figure 4B, Kaplan–Meier survival analysis demonstrated that the 5-year survival rate of RCC patients with higher GADD45A tumor expression (79% of the 5-year survival rate) was significantly better than that of patients with low expression (63% of the 5-year survival rate, *p* < 0.001). The mechanisms by which NPS-1034 treatment affects apoptosis and TNFRSF1A signaling regulation in RCC cells were investigated via TNFRSF1A overexpression transfection (Figure 4C). Promoting GADD45A expression may subsequently activate downstream proteins to inhibit cell viability and lead to apoptosis. Conversely, inhibiting the IκBα/NFκB signaling pathway may suppress downstream proteins and be associated with apoptotic effects. However, the elevated GADD45A and decreased IκBα/NFκB-related pathway expression results were significantly reversed via TNFRSF1A overexpression. As a result, NPS-1034 suppressed proliferation and ameliorated apoptosis via the GADD45A and TNFRSF1A signaling pathways.

### 3.7. NPS-1034 Suppressed Tumor Growth in an In Vivo Renca-Luc Model

**Figure 5:**
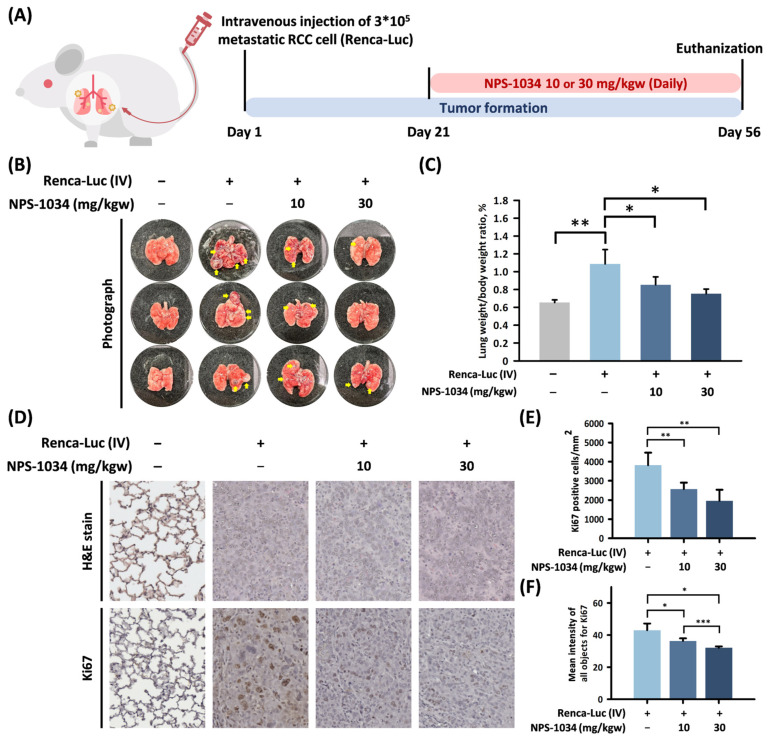
NPS-1034 alleviated the tumor burden and inhibited cell proliferation in a lung-metastatic animal model. (**A**) Flow chart and schematic overview of the Renca-luc metastatic animal model in vivo. (**B**) Representative image of gross lung specimens. The yellow arrow marks the site of tumor formation. (**C**) Mice lung weight normalized by body weight. Data are shown as mean ± S.D. (* *p* < 0.05; ** *p* < 0.01; *** *p* < 0.001) (**D**) Overview of pathological changes and the rate of cell proliferation in the tumor were analyzed by H&E and Ki-67 staining. (**E**,**F**) Estimation of tumor burden by determining Ki-67-positive cells and mean intensity of all objects for Ki-67. Data are shown as mean ± S.D. (* *p* < 0.05; ** *p* < 0.01; *** *p* < 0.001).

An RCC tumor model was established in BALB/c mice via intravenous injection of 1 × 10^5^ Renca-luc cells through the tail vein (Figure 5A). The in vivo Renca-luc model predominantly metastasized to the lung, allowing us to evaluate tumor progression and organ tumor burdens. NPS-1034 was orally administered daily for 5 weeks with 10 or 30 mg/kgw beginning on day 21. The gross morphology of the lung is shown in Figure 5B. The number and volume of tumor particles in the non-treatment group were significantly higher than those in the administration group. The number of tumor particles in the high-dose group (30 mg/kgw) was more sporadic than in the lower-dose group (10 mg/kgw). Furthermore, the lung–body weight ratio (Figure 5C) showed that when the NPS-1034 dose increased, it markedly inhibited tumor growth, and the proportion of lung tumors to body weight decreased. Compared with the non-treatment group (1.09% lung/body weight ratio), the 10 mg/kgw treatment for the NPS-1034 group was 0.85%, and for 30 mg/kgw, it was 0.75 (*p* < 0.03 and *p* < 0.02, respectively), which were significantly decreased values.

To assess the effects of NPS-1034 on the pathological changes and proliferation rate in the Renca-luc tumor, H&E staining and prognostic marker Ki-67 staining were used to explore the tumor histologic sections, as shown in Figure 5D. The tumor burden was estimated by determining the number of Ki-67-positive cells and the mean intensity of all Ki-67 staining (Figure 5E,F). The number of Ki67-positive cells significantly decreased as the dose administration of NPS-1034 increased (2574.88 and 1963.31 positive cells/mm^2^, *p* < 0.01 and *p* < 0.003, respectively) compared with the non-treatment group (3813.88 positive cells/mm^2^). The mean intensity of all staining for Ki-67 significantly decreased when the concentration of NPS-1034 treatment increased (36.4 and 32.1, *p* < 0.025 and *p* < 0.012, respectively) compared with the non-treatment group (43.0). Meanwhile, the high-dose and lower-dose groups both significantly decreased (*p* < 0.001).

## 4. Discussion

To the best of our knowledge, this is the first study to elucidate the antitumor role of NPS-1034 in RCC in vitro and in vivo using the Renca-luc metastatic animal model. Our findings confirmed that NPS-1034 inhibits the MET, AXL, and related pathways. In addition, we assessed the prognosis evaluation factors of GADD45A- and TNFRSF1A-related pathways via an apoptosis array and GO and KEGG enrichment analysis and found that they were related to cell proliferation, differentiation, and even cell death activation, including apoptosis. The elevated GADD45A-related pathway expression results and the decreased TNFRSF1A-related pathway expression results were reversed by TNFRSF1A overexpression. Nevertheless, we found that apoptosis not only played a crucial role in NPS-1034-induced RCC death in vitro but also in vivo. Our findings show that increasing NPS-1034 treatment doses in vivo in the Renca-luc metastatic animal model markedly inhibits tumor growth and the proportion of lung–body weight; in addition, the morphology of the number and volume of tumor metastases decreased. Suppressing Ki67 expression demonstrated the significant effect of NPS-1034 treatment in an in vivo metastatic animal model. Our study sheds new light on this study area and highlights the potential value of alternative adjuvant therapy in RCC.

In the present study, apoptosis played a crucial role in RCC cell death when NPS-1034 was applied in vitro. The proapoptotic effects of NPS-1034 were assessed via flow cytometry and fluorescence microscopy Hoechst 33342 staining (Figure 1F–I). Morphological changes were observed in the nuclei of RCC cells, further confirming that NPS-1034 induced cell death by promoting cell apoptosis. Additionally, our findings follow previous results showing that NPS-1034 plays a crucial regulatory role as an inhibitor in both the MET and AXL pathways in solid tumors, such as lung cancer [23,34] and gastric cancer [30]. However, by using the human Proteome Profiler™ Antibody Array and next-generation sequencing (NGS), GO and KEGG enrichment analysis was able to play significant roles in further implicating the regulatory role in GADD45A- and TNFRSF1A-related pathways in the mechanism underlying NPS-1034 treatment in RCC (Supplement Figure S1). An increase in GADD45A expression may subsequently activate downstream proteins, inhibiting cell viability and leading to apoptosis [35]. Moreover, in terms of the 5-year survival rate, we found that RCC patients with higher GADD45A tumor expression had significantly better outcomes than those with lower expression, further implicating the potential of GADD45A as a predictor of outcomes in RCC patients. Additionally, in our previous study of NPS-1034 treatment in testicular cells, we found that TNFRSF1A plays a crucial regulatory role in promoting apoptosis via the IκBα/NFκB signaling pathway [31] (Figure 3). The present study provided evidence supporting the link between GADD45A and TNFR1 via TNFRSF1A overexpression since TNFRSF1A overexpression significantly reversed the effects of NPS-1034 on the GADD45A- and TNFR1-related pathways. These results support the hypothesis that NPS-1034’s mechanism of action on RCC is mediated via the GADD45A- and TNFR1-related pathways (Figure 4).

Additionally, the present study demonstrated that NPS-1034 has strong anticancer efficacy in vivo (Figure 5). We employed a Renca-luc mouse model, which predominantly metastasizes to the lung, to identify and evaluate tumor progression and organ tumor burdens. Previous studies have established that the Renca-luc model demonstrates efficient lung metastasis within a predictable timeframe [36,37]. To focus on the effects of NPS-1034 in reducing cancer cell proliferation and increasing cancer cell apoptosis, we optimized the in vivo dose and treatment schedule. Starting on the 21st day postinjection of Renca-luc cells, NPS-1034 was used for daily treatments. After oral treatment with NPS-1034 for 5 weeks, the number and volume of tumor particles markedly decreased compared with the non-treatment group. The pathological changes in H&E staining and decreased Ki-67 staining in the administration group further indicated a decreased tumor cell proliferation rate.

However, choosing representative cell lines for RCC research is difficult since there are different types of RCC according to their pathology [38]. Most patients suffer from ccRCC, but patients with certain non-ccRCC types might have worse prognoses. The WHO 2022 classification of kidney tumors groups these pathological characteristics into “clear cell renal tumors”, “papillary renal tumors”, “oncocytic and chromophobe renal tumors”, and “collecting duct tumors” and adds two categories: “other renal tumors” and “molecularly defined renal carcinomas”. However, it is still difficult to clearly distinguish these subtypes [39]. For cell lines, in this study, we chose those more likely to present ccRCC characteristics [38], and this is a limitation regarding our results, since they may not represent all RCC types.

**Figure 6:**
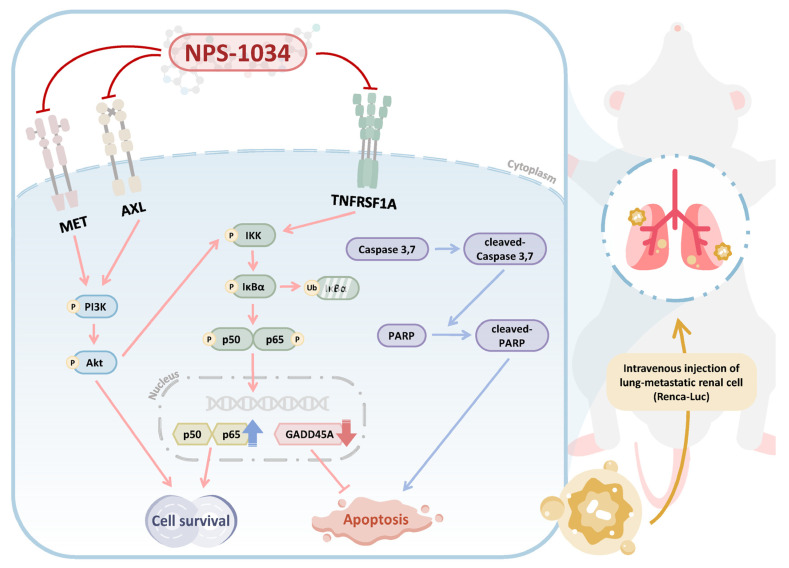
Schematic overview of NPS-1034-targeted pathways in RCC. Our results showed that NPS-1034 not only inhibits the known AXL and MET pathways but also inhibits the TNFRSF1A downstream signaling pathway and activates the GADD45A pathway. The favorable anticancer efficacy was also demonstrated in vivo in the Renca-luc metastatic animal model.

Notably, our study has some limitations. Although three representative RCC cell lines were used, it is still not possible to fully represent all RCC cell lines. Moreover, despite using the animal model presented, further confirmation is needed regarding the differences caused by different orientations in the sections. Thus, we presented the sections that show significant differences in the pathological changes and proliferation rate of the NPS-1034 treatment in tumors. Finally, the findings may not fully represent the complexities and variations observed in real-world human RCC cases. This study lacked clinical data from human patients, making it challenging to directly relate the findings to real patient outcomes. Clinical trials and studies involving human subjects are necessary to validate the efficacy and safety of NPS-1034. In summary, our study revealed the anticancer efficacy of NPS-1034 in RCC. With in vivo validation, the Renca-luc metastatic animal model demonstrated the regulatory role of NPS-1034 treatments in RCC via GADD45A- and TNFRSF1A-related pathways (Figure 6).

## Data Availability

All analyzed data are included in this article. Additional information is available upon request.

## Supplementary Materials

The following supporting information can be downloaded at https://www.mdpi.com/article/10.3390/cells13201713/s1: Supplement Figure S1. Determination of apoptosis-related protein expression altered by NPS-1034 treatment. (A) A498, 786-0, and Caki-1 cells treated with NPS-1034 (0 and 80 μM) for 48 h were analyzed using the human Proteome Prolifer™ Antibody Array. (B) Quantitative analysis showing different changes in apoptosis-related markers in A498, 786-0, and Caki-1 cells.

## Abbreviations

ccRCC: clear cell RCC; DMSO: dimethyl sulfoxide; GADD45A: growth arrest and DNA damage-inducible alpha; GO: gene ontology; H&E: hematoxylin and eosin; KEGG: Kyoto Encyclopedia of Genes and Genomes; PBS: phosphate-buffered saline; PI: propidium iodide; RCC: renal cell carcinoma; RIPA: radioimmunoprecipitation; RTKs: receptor tyrosine kinases; SDS-PAGE: sodium dodecyl sulfate–polyacrylamide gel electrophoresis; TKI: tyrosine kinase inhibitor; TNFRSF1A: TNF receptor superfamily member 1A; VEGF: vascular endothelial growth factor; VEGFRs: vascular endothelial growth factor receptors; VHL: von Hippel–Lindau; NGS: next-generation sequencing.
