# Supplementary material for: NPS-1034 Exerts Therapeutic Efficacy in Renal Cell Carcinoma Through Multiple Targets of MET, AXL, and TNFRSF1A Signaling in a Metastatic Model
Source: Cells. 2024 Oct 17;13(20):1713. doi: 10.3390/cells13201713 (PMC11506434; doi:10.3390/cells13201713)
Supplement: Supplementary file 1 [file cells-13-01713-s001.zip › cells-3122885-supplementary.pdf]

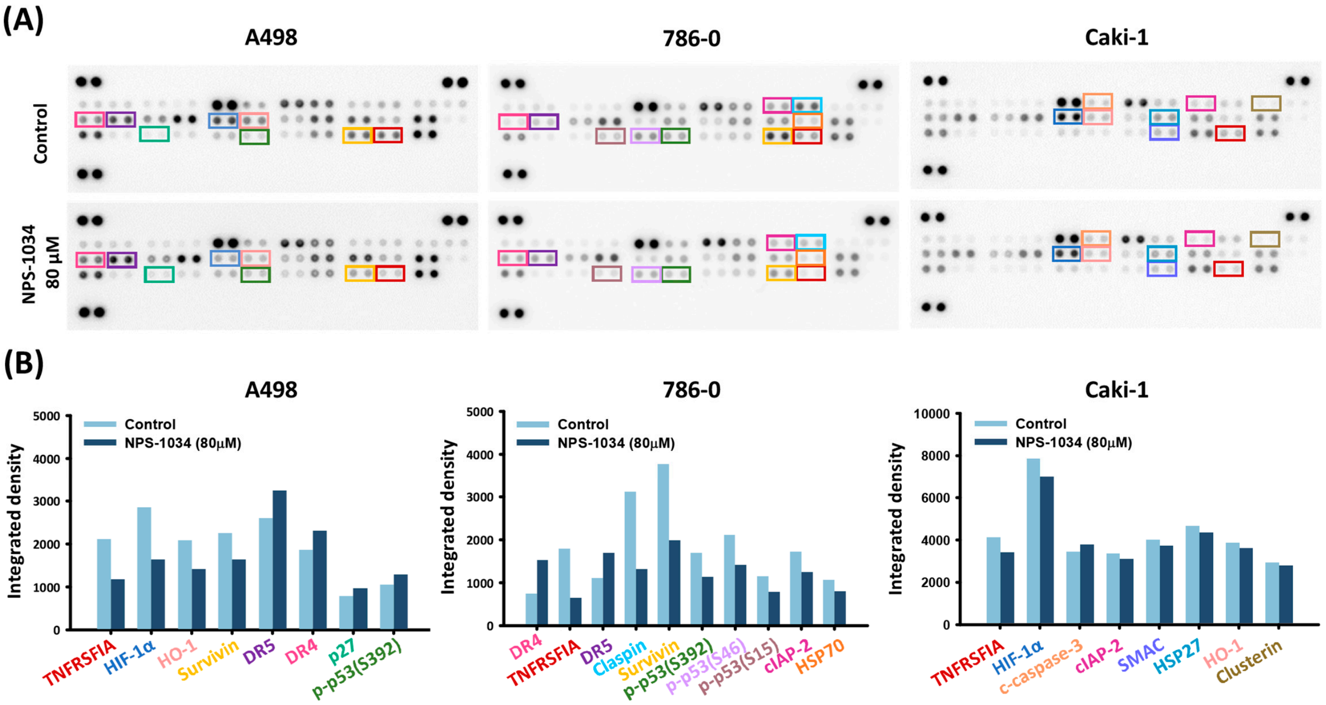

Supplementary Figure S1. Determination of apoptosis-related protein expression altered by NPS-1034 treatment. (A) A498, 786-0, and Caki-1 cells treated with NPS-1034 (0 and 80 μM) for 48 h were analyzed using the human Proteome Prolifer™ Antibody Array. **(B)** Quantitative analysis showed different changes in apoptosis-related markers in A498, 786-0, and Caki-1 cells.
